# Preclinical Activity of Datopotamab Deruxtecan, an Antibody–Drug Conjugate Targeting Trophoblast Cell-Surface Antigen 2, in Uterine Serous Carcinoma

**DOI:** 10.1158/2767-9764.CRC-25-0057

**Published:** 2025-05-09

**Authors:** Michelle Greenman, Cem Demirkiran, Stefania Bellone, Tobias M.P. Hartwich, Blair McNamara, Victoria M. Ettorre, Niccolo G. Santin, Namrata Sethi, Yang Yang-Hartwich, Katyayani Papatla, Elena Ratner, Alessandro D. Santin

**Affiliations:** Department of Obstetrics, Gynecology, and Reproductive Sciences, Yale University School of Medicine, New Haven, Connecticut.

## Abstract

Uterine serous carcinoma (USC) is a rare subset of endometrial cancer with a poor prognosis and high recurrence rate. Datopotamab deruxtecan (Dato-DXd) is a novel antibody–drug conjugate (ADC). The objective of this study was to evaluate the preclinical activity of Dato-DXd in USC *in vitro* against primary USC cell lines with various trophoblast cell-surface antigen 2 (TROP2) expression and *in vivo* in TROP2-overexpressing cell line–derived mice xenografts. USC primary tumor cell lines were treated with Dato-DXd and a control ADC (CTL ADC) to evaluate cell viability following exposure. Antibody-dependent cell-mediated cytotoxicity against TROP2-overexpressing and -nonexpressing cell lines was evaluated using a 4-hour chromium release assay. USC xenografts in mice were treated with Dato-DXd, CTL ADC, datopotamab, and vehicle to assess the *in vivo* effects via retro-orbital Dato-DXd administration. We found USC cell lines with TROP2 overexpression to be significantly more sensitive to killing induced by Dato-DXd compared with CTL ADC *in vitro* (e.g., IC_50_: 0.11 µmol/L vs. 30.07 µmol/L, *P* = 0.0074 and 0.11 µmol/L vs. 48.95 µmol/L, *P* = 0.0127, respectively). Dato-DXd induced antibody-dependent cell-mediated cytotoxicity in the presence of peripheral blood lymphocytes from healthy donors. TROP2-nonexpressing cell lines demonstrated minimal killing by Dato-DXd; however, when admixed with TROP2-overexpressing cells, a significant bystander effect was appreciated. *In vivo*, mice xenografts overexpressing TROP2 treated with Dato-DXd demonstrated tumor growth suppression and longer overall survival compared with CTL ADC–treated xenografts. These data demonstrate Dato-DXd to be highly active against TROP2-overexpressing USC *in vitro* and *in vivo*. Our preclinical activity results warrant future clinical trials for patients with advanced or recurrent USC.

**Significance:**

Targeted treatment of USC using the biomarker TROP2 represents a significant opportunity for further treatment options for patients already resistant to other lines of treatment. In this study, we present data showing preclinical evidence of effectiveness of this biomarker-targeted therapy in USC.

## Introduction

In the United States, endometrial cancer is the most common gynecologic malignancy with an estimated 66,200 new cases in 2023 (1). Despite the high incidence, the 5-year relative survival is approximately 81% (1). Uterine serous carcinomas (USC) are a rare subset that make up approximately 10% of all endometrial cancers and generally have a worse prognosis and higher risk of relapse even when diagnosed at earlier stages (2). Although USC makes up a small proportion of endometrial cancer cases, it accounts for 39% of disease-specific deaths (3). Standard-of-care treatment involves cytoreductive surgery, and given the high propensity for recurrence, platinum- and taxane-based systemic treatment plus brachytherapy (4). Understanding of the molecular landscape of USC has led way to tailored therapy, most notably targeting HER2. Given the high rates of recurrent or metastatic disease, further development of novel, targeted therapy is critical.

Antibody–drug conjugates (ADC) are a newer growing pharmaceutical class composed of a highly specific mAb conjugated by a linker to a potent cytotoxic payload (5). The payload systems are either antimicrotubule compounds or DNA-damaging agents, which lead to cell-cycle arrest and apoptosis (6). The ADC technology involves the selection of a linker that remains stable in circulation in order to prevent off-target effects (7). Linker design plays a paramount role as premature release of the payload into circulation can lead to systemic toxicity (5). Linkers can be cleavable or noncleavable in design, with noncleavable linkers having the advantage of prolonged serum stability and efficient release into tumor cells (8). Following clathrin-mediated endocytosis, tumor cell lysosomes rapidly cleave the linker between the antibody and payload, resulting in the release of the cytotoxic agent (9). Additionally, the use of a cleavable linker facilitates drug permeability into surrounding cells, inducing killing, regardless of the expression of the ADC target antigen, also known as the bystander antitumor effect (10). Mirvetuximab soravtansine targeting folate receptor α in platinum-resistant ovarian cancer, tisotumab vedotin targeting tissue factor in recurrent and metastatic cervical cancers, and trastuzumab deruxtecan in all HER2-positive unresectable solid tumors exemplify successful ADCs that have entered into the treatment of gynecologic malignancies (11–13).

Trophoblast cell-surface antigen 2 (TROP2) is a transmembrane glycoprotein composed of an extracellular domain, a transmembrane region and a short cytoplasmic tail that can be phosphorylated (14). The transmembrane domain acts as a calcium signal transducer altering cell cycle–related signaling pathways (14). Binding of TROP2 to various proteins such as insulin-like growth factor 1, claudin-1 and -7, cyclin D1, and protein kinase C leads to multiple downstream effects responsible for cell proliferation and apoptosis (15). The cytoplasmic tail serves in regulating protein–protein interactions, and its protein kinase C phosphorylation site allows for the modulation of TROP2 in calcium signaling (16). TROP2 is commonly overexpressed in cancers with poor prognosis, making it an excellent target for cancer therapy (17). The low level expression of TROP2 in healthy tissues and reports of up to 95% expression in USC make it a promising target for cancer therapy (18).

Datopotamab deruxtecan (Dato-DXd) is a novel TROP2-directed ADC. The ADC consists of the recombinant humanized anti-TROP2 IgG1 mAb conjugated by a tetrapeptide-based cleavable linker to a topoisomerase I (Topo I) inhibitor (DXd; ref. 19). Dato-DXd binds with high affinity to cell-surface TROP2 and is internalized by tumor cells, and then the tetrapeptide linker is cleaved with lysosomal enzymes, leading to the release of DXd (19). Subsequent DNA damage and resulting apoptosis are induced by DXd-mediated Topo I inhibition (20, 21).

Dato-DXd was approved in January 2025 by the FDA for use in unresectable or metastatic, hormone receptor–positive, and HER2-negative breast cancers. The results from the TROPION-PanTumor01 study show a progression-free survival (PFS) HR of 0.63 (*P* < 0.0001) and a trend in overall survival (OS) with a HR of 0.84 for patients that received Dato-DXd compared with eribulin/vinorelbine/capecitabine/gemcitabine with hormone receptor–positive breast cancer and HER2-negative breast cancer (22). In patients heavily pretreated with advanced/metastatic non–small cell lung cancer, phase III trial data showed improved PFS and a trend toward improved OS but did not reach statistical significance (23).

The objective of this study was to evaluate the efficacy of Dato-DXd in USC primary cell lines and cell line–derived xenograft (CDX) models with variable TROP2 expression. We present for the first time preclinical data *in vitro* and *in vivo* that Dato-DXd is highly effective in TROP2-overexpressing USC. Taken together, our results suggest that Dato-DXd may have clinical activity against TROP2-overexpressing USC tumors.

## Materials and Methods

### Establishment of cell lines

The approval for this study was obtained through the Institutional Review Board. All patients were consented via written informed consent before tissue/blood collection per institutional guidelines and the Declaration of Helsinki. Eleven primary USC cell lines established from chemotherapy-naïve patients at the time of primary staging surgery, *Mycoplasma*-free and with limited number of *in vitro* passages (i.e., less than 50), were used in our experiments. Primary tumor cell lines were established and authenticated using whole-exome sequencing, as previously described (24), and then deidentified. Cell sample characteristics and patient demographics are described in Table 1. Tumors were staged according to the International Federation of Gynecology and Obstetrics staging system.

**Table 1:** Characteristics and demographics of USC cell lines

Cell line	Age	Race	International Federation of Gynecology and Obstetrics	Histology	TROP2 MFI	Flow cytometry TROP2 MFI ± SD	Score
ARK1	62	Black	IVA	USC	0	13.88 ± 17.49	0
ARK2	63	Black	IVB	USC	90.12	125.28 ± 150.01	3+
ARK4	73	White	IVB	USC	1.76	18.71 ± 17.90	0
ARK6	62	White	IB	Mixed USC	39.02	56.28 ± 81.0	2+
ARK7	75	White	IIC	USC	17.25	26.15 ± 18.20	1+
ARK11	80	Black	IIIC	Mixed USC	7.12	35.61 ± 25.14	1+
ARK19	65	White	IA	USC	162.07	213.8 ± 170.73	3+
ARK20	42	White	II	USC	86.41	148.65 ± 151.03	3+
ARK22	67	White	IV	USC	49.5	75.6 ± 37.14	2+
ARK24	54	Black	IIIC	Mixed USC	31.37	55.07 ± 37.98	2+
ARK26	68	White	IVB	USC	18.24	40.45 ± 34.69	1+

### TROP2 expression analysis

Primary USC cell lines were analyzed by flow cytometry for TROP2 expression by incubating single-cell tumor suspensions with 2.5 µg/mL of primary antibody hRS7 IgG for 30 minutes at 7°C. A FITC–conjugated goat anti-human F(ab1)2 Ig was used as a secondary antibody (BioSource International). Data were then acquired on CellQuest software (BD Biosciences; RRID: SCR_014489) using a FACScalibur. The mean fluorescence intensity (MFI) was evaluated using CellQuest (BD Biosciences) and Prism 8. Cell lines with a MFI >100 were determined to have 3+ expression of TROP2, whereas cell lines with an MFI of 51 to 100 were noted to have 2+, 21 to 50, 1+, and 20 or less considered 0.

### Drug

Dato-DXd, nontargeting control ADC (CTL ADC), and unconjugated datopotamab antibody (TROP2 IgG1 mAb, datopotamab) were obtained from Daiichi Sankyo Co., Ltd. through a Material Transfer Agreement. Dato-DXd and CTL ADC were stored at −80°C until use. Dato-DXd is a humanized mAb against TROP2, covalently linked with a potent DNA Topo I inhibitor by applying the DXd-ADC technology platform with the optimized drug-to-antibody ratio (DAR) of 4 (19). The agents were reconstructed using the solvent, and serial dilutions were prepared using a vehicle solution (10 mmol/L acetate buffer and 5% sorbitol, pH 5.5) to create the final concentrations ranging from 0.01 to 50 µg/mL for *in vitro* evaluation. For *in vivo* studies, the drug was prepared into a stock weekly with a vehicle of polyethylene glycol 400, water, and 100% ethanol and kept at 4°C shielded from light to ensure a dose of 10 mg/kg in each retro-orbital injection.

### Flow cytometry–based cell viability assay

USC cell lines were plated in six-well tissue culture plates at a density of 70,000 to 80,000 cells/well in RPMI 1640 media supplemented with 10% FBS, 1% penicillin/streptomycin, and 1% amphotericin. Cells were incubated at 37°C, with 5% CO_2_ for 24 hours. After overnight incubation, nonsuspended cells were washed off with PBS, and 2 mL of tissue culture media were added to each culture well. Cells were then treated with Dato-DXd at concentrations of 0.01, 0.1, 0.5, 5.0, and 50 μg/mL. The treated cells were then allowed to incubate for 72 hours. After 72 hours, well contents were harvested in their entirety, centrifuged, and stained with propidium iodide (PI; 2 µL of 500 µg/mL stock solution in PBS). The viable cells were then quantified using flow cytometry as a mean ± SEM relative to untreated cells as 100% viable controls. A minimum of three independent experiments per cell line was performed to determine the IC_50_ value of Dato-DXd in cancer cell lines.

### Bystander antitumor effect assay

Bystander antitumor effect assays were performed by admixing TROP2 0 cells (i.e., ARK4) stably transfected with GFP plasmid (pCDH-CMV-MCSEF1-copGFP, donated by Dr. Simona Colla, M.D. Anderson Cancer Center, Houston, Texas), with TROP2 3+ cells [i.e., ARK2 in a 1:1 ratio (20,000 cells/well of each cell type)]. Cells were placed in six-well plates, and after overnight incubation, cells were treated with Dato-DXd or CTL ADC at a concentration of 0.5 μg/mL. After 48 hours of incubation, cells were harvested and washed with PBS and stained with PI (2 µL of 500 µg/mL stock solution in PBS). The analysis of cell viability after treatment was performed using a flow cytometry–based assay, which allowed us to quantify the fluorescently tagged viable ARK4 cells versus the nontagged ARK2 cells. Using the cell viability data acquired through PI staining, we were then able to quantify the percentage of viable cells as a mean ± SEM relative to untreated cells as 100% viable controls for the ARK2 cell line. A minimum of three independent experiments were performed.

### Antibody-dependent cell-mediated cytotoxicity

Standard 4-hour chromium (^51^Cr) release assay was performed to measure the cytotoxic reactivity of Ficoll-Hypaque–separated peripheral blood mononuclear cells from several healthy donors in combination with datopotamab, Dato-DXd, or CTL ADC against ^51^Cr-labeled primary USC target cell lines with high and low TROP2 expression at effector-to-target ratios of 5:1 and 10:1, respectively. The release of ^51^Cr from target cells was measured as evidence of tumor cell lysis after exposure of the tumor cells to 2.5 µg/mL of rituximab (anti-CD20), datopotamab, CTL ADC, or Dato-DXd. As a positive control condition, 0.1% SDS was used to achieve complete lysis of target cells. Chimeric anti-CD20 mAb rituximab 2.5 µg/mL was used as the negative control for datopotamab, Dato-DXd, or CTL ADC in all bioassays. The percentage cytotoxicity of each drug was calculated by the following formula: % cytotoxicity = 100 × (E − S)/(T − S), in which E is the experimental release, S is the spontaneous release by target cells, and T is the maximum release by target cells lysed with 0.1% SDS. A minimum of three replicates was performed. Different donor peripheral blood lymphocytes (PBL) were used for each replicate.

### Double-stranded DNA breakage assay

To detect double-stranded DNA breaks, ARK2 cells were stained with a PE Mouse Anti-H2AX (pS139) antibody (BD Biosciences, catalog number 562377). The cells were incubated with 0, 5, and 50 μg/mL of vehicle, CTL ADC, or Dato-Dxd for 72 hours and then harvested and collected. Briefly, the cells were resuspended in 200 μL of fixative buffer (4% paraformaldehyde in PBS 1X) and incubated for 15 minutes at room temperature. The cells were washed once with PBS and were permeabilized using 200 μL of a solution of PBS with 0.5% saponin and 1% BSA. The PE Mouse Anti-H2AX was added to the cells, 5 μL per sample, and incubated on ice for 45 minutes. Cells were washed twice with a solution of PBS with 0.5% of BSA. Cells were analyzed for the phosphorylation of H2AX using a BD FACSCalibur (RRID: SCR_000401).

### *In vivo* experiments

The *in vivo* antitumor activity of Dato-DXd and CTL ADC was tested in mouse CDX models with TROP2 3+ expression. The ARK2 USC cell line was chosen for the CDXs as it has high TROP2 expression and has previously shown to be able to consistently grow as xenografts in SCID mice (25, 26). The cell line was injected into 6- to 8-week-old SCID mice subcutaneously (Harlan Laboratories). Each mouse was injected with 7 million uterine cancer cells suspended in approximately 200 µL of a 1:1 solution of sterile PBS-containing cells and Matrigel (BD Biosciences). After the tumor volume reached 0.2 cm^3^, the mice were randomized into four treatment groups: Dato-DXd (10 mg/kg), CTL ADC (10 mg/kg), datopotamab (10 mg/kg), and vehicle, with five animals per study group. *In vivo* dosages were chosen based on data published on the bioavailability of the compound in preclinical mouse models (19). All treatment drugs were given as a single retro-orbital injection on day 0. Mice were observed for OS as the primary outcome measure. Tumor volume was measured twice weekly. Tumor volume was determined using the formula (A^2^ × B)/2, in which B represented the largest tumor diameter size and A was the smaller perpendicular tumor diameter. Mice were euthanized if tumor volume reached 1.0 cm^3^, as tumor growth exceeding this size was considered inhumane per our Institutional Animal Care and Use Committee (IACUC) protocol. At the conclusion of the study on day 50, the surviving mice were euthanized. Animal care and euthanasia were carried out according to the rules and regulations set forth by the IACUC. This study was approved by the IACUC.

### Statistical analysis

Statistical analysis was performed using GraphPad Prism 7 (GraphPad Software, Inc; RRID: SCR_002798). The differences in the inhibition of proliferation/killing in the uterine cancer cell lines after exposure to Dato-DXd, CTL ADC, and datopotamab, the bystander assays, and the antibody-dependent cell-mediated cytotoxicity (ADCC) experiments were evaluated by two-tailed unpaired Student *t* test. One-way ANOVA was used to evaluate the significant differences in tumor volumes at specific time points in the *in vivo* experiments. OS data (OS defined as the time of enrollment to either death or tumor volume of 1.0 cm^3^) were analyzed and plotted using Kaplan–Meier survival curves, which were compared for differences using the log-rank test. Differences in all comparisons *P* < 0.05 were considered statistically significant.

### Data availability

The data generated in this study are available upon request from the corresponding author.

## Results

### TROP2 expression in primary USC cell lines

Flow cytometry was used to evaluate the expression of the TROP2 protein among the 11 primary USC cell lines. TROP2-high expression (3+) was verified in three of the 11 lines (27.3%; Table 1). The additional three cell lines were expressed at the 2+ level (27.3%). The remaining six cell lines had low to negligible expression (i.e., 0 to 1+). Given these results, two cell lines with TROP2 overexpression were selected, ARK2 and ARK20, for the following described *in vitro* and *in vivo* experiments. ARK7 was selected as a low expressor (TROP2 1+). An additional nonexpressing cell line, ARK1 (TROP2 0), was used in experiments as a negative control.

### Cell viability experiments with Dato-DXd *in vitro*

**Figure 1:**
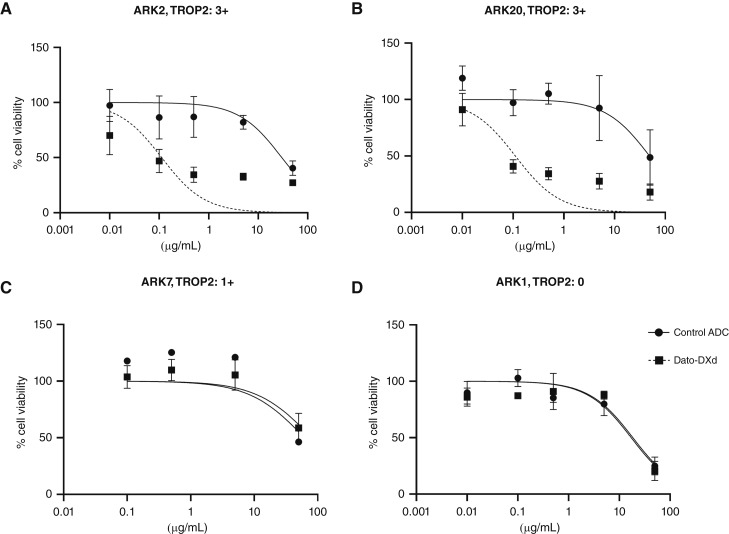
IC_50_ value dose–response curves of Dato-DXd and CTL ADC in USC cell lines with variable TROP2 expression. **A,** TROP2 3+ ARK2 USC cell line: the mean IC_50_ value of Dato-DXd was 0.11 μg/mL vs. 30.07 μg/mL for CTL ADC (*P* = 0.0074). **B,** TROP2 3+ ARK20 USC cell line: the mean IC_50_ value of Dato-DXd was 0.11 μg/mL vs. 48.95 μg/mL for CTL ADC (*P* = 0.0127). **C,** TROP2 1+ ARK7 USC cell line: the mean IC_50_ value of Dato-DXd was 80.38 μg/mL vs. 61.79 μg/mL for CTL ADC. **D,** TROP2 ARK1 USC cell line: the mean IC_50_ value was 18.49 μg/mL vs. 17.12 μg/mL for CTL ADC (*P* = 0.65).

The chosen cell lines (Table 1) were treated with scalar concentrations of Dato-DXd or CTL ADC ranging from 0.01 to 50 μg/mL with a subsequent incubation of 72 hours. In both TROP2 3+ USC cell lines, Dato-DXd was significantly more effective at inducing cell death than the CTL ADC (Fig. 1A and B). In ARK2 (TROP2 3+), the mean IC_50_ values for Dato-DXd and CTL ADC were 0.11 μg/mL and 30.07 μg/mL, respectively (*P* = 0.0074). In ARK20 (TROP2 3+), the mean IC_50_ values for Dato-DXd and CTL ADC were 0.11 and 48.95 μg/mL, respectively (*P* = 0.0127). In ARK7, the low-expressing cell line (TROP2 1+), there was no significant difference in cell death with either Dato-DXd or CTL ADC (*P* = 0.32; Fig. 1C). In ARK1, the selected nonexpressing cell line (TROP2 0), there was no significant difference in induced cell death observed following the same incubation period (mean IC_50_ value of 18.49 μg/mL with Dato-DXd and 17.12 μg/mL with CTL ADC, *P* = 0.6535; Fig. 1D). The datopotamab-unconjugated (i.e., “naked”) antibody did not reach an IC_50_ value when used against any of the primary cell lines tested regardless of their expression of TROP2.

### Bystander antitumor effect *in vitro*

**Figure 2:**
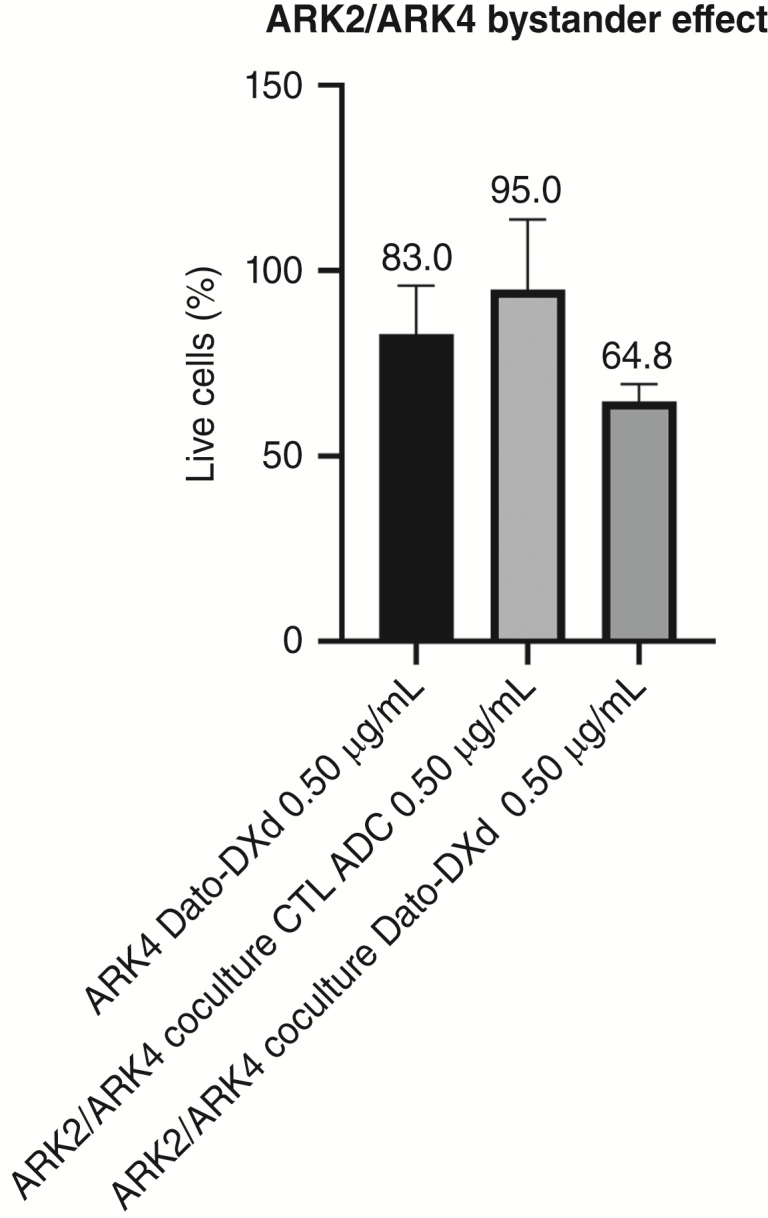
Bystander antitumor effect of TROP2-nonexpressing USC cells (ARK-4) when cocultured with TROP2 3+ USC ARK2 cell line treated with 0.5 μg/mL of Dato-DXd vs. CTL ADC. Dato-DXd had little effect on ARK-4 when they were cultured alone (83% lives cells); however, when ARK-4 was cocultured with TROP2 3+ ARK2, Dato-DXd induced significantly more ARK-4 cell killing (64.8% live cells, *P* = 0.0231). CTL ADC did not have significant cell killing with coculture (95% live cells).

Next, we evaluated the ability of Dato-DXd and CTL ADC to induce bystander antitumor effect when TROP2 3+ USC cells (ARK2) were admixed with negligible TROP2 expression cancer cells (ARK4) for an incubation period of 48 hours. As demonstrated in Fig. 2, the ARK2/ARK4 cocultures exemplified greater bystander antitumor effect of TROP2-nonexpressing ARK4 cells following treatment with Dato-DXd than when treated with CTL ADC (i.e., ARK4 treated with Dato-DXd had 83% live cells, whereas ARK2/ARK4 coculture treated with Dato-DXd had 64.8% live cells, *P* = 0.0231; Fig. 2). When TROP2-nonexpressing ARK4 cells cocultured with ARK2 were challenged with CTL ADC, a minimal, nonsignificant bystander antitumor effect was detected (95% live cells).

### Dato-DXd– and datopotamab-mediated ADCC against TROP2-overexpressing primary USC cell lines

**Figure 3:**
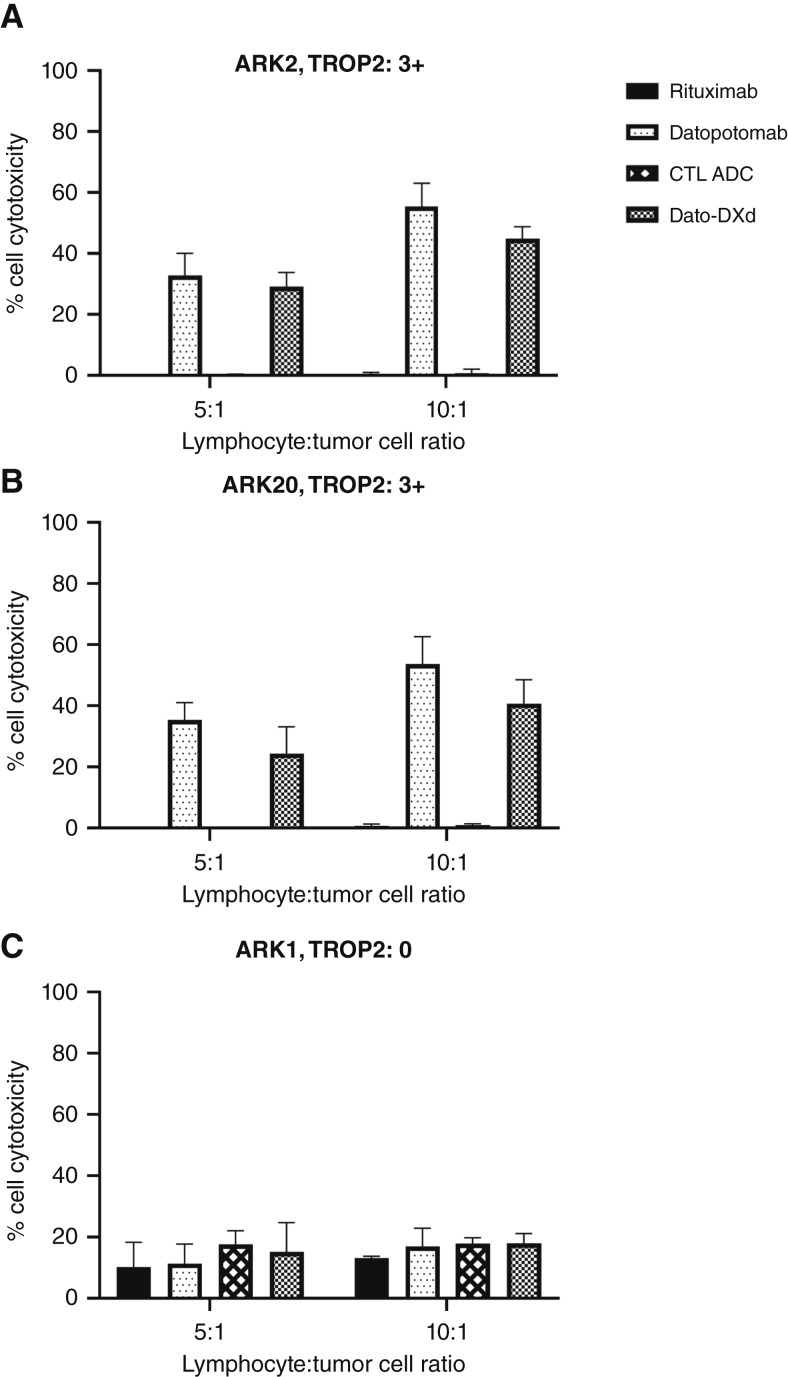
ADCC results of Dato-DXd, datopotamab, rituximab, and CTL ADC in representative (**A**) ARK2 USC TROP2 3+, (**B**) ARK20 USC TROP2 3+, and (**C**) ARK1 USC TROP2-nonexpressing cell lines.

The selected USC cell lines were tested for their sensitivity to ADCC when challenged with heterologous PBLs from healthy donors in standard 4-hour^51^Cr release assays. When combined with PBLs and rituximab (2.5 μg/mL), all USC cell lines did not experience significant cytotoxicity. Both Dato-DXd and datopotamab (unconjugated antibody) were effective at inducing cytotoxicity in TROP2-overexpressing cell lines ARK2 and ARK20 (Fig. 3A and B). For ARK2, the mean cytotoxicity ± SEM of Dato-DXd was 29.1 ± 2.7% for ratio 5:1 and 44.9 ± 2.3% for ratio 10:1 and for datopotamab was 32.8 ± 4.2% for ratio 5:1 and 55.4 ± 4.4% for ratio 10:1 (*P* < 0.0001). In ARK20, the mean cytotoxicity for Dato-DXd was 24.4 ± 5.1% for ratio 5:1 and 40.7 ± 5.1% for ratio 10:1 and for datopotamab was 35.1 ± 3.3% for ratio 5:1 and 53.7 ± 5.1% for ratio 10:1 (*P* < 0.0001). In ARK1, the TROP2-nonexpressing cell line, negligible killing was observed following exposure to both Dato-DXd and datopotamab (Fig. 3C).

### *In vitro* double-stranded DNA break assay after treatment with Dato-DXd or CTL ADC

**Figure 4:**
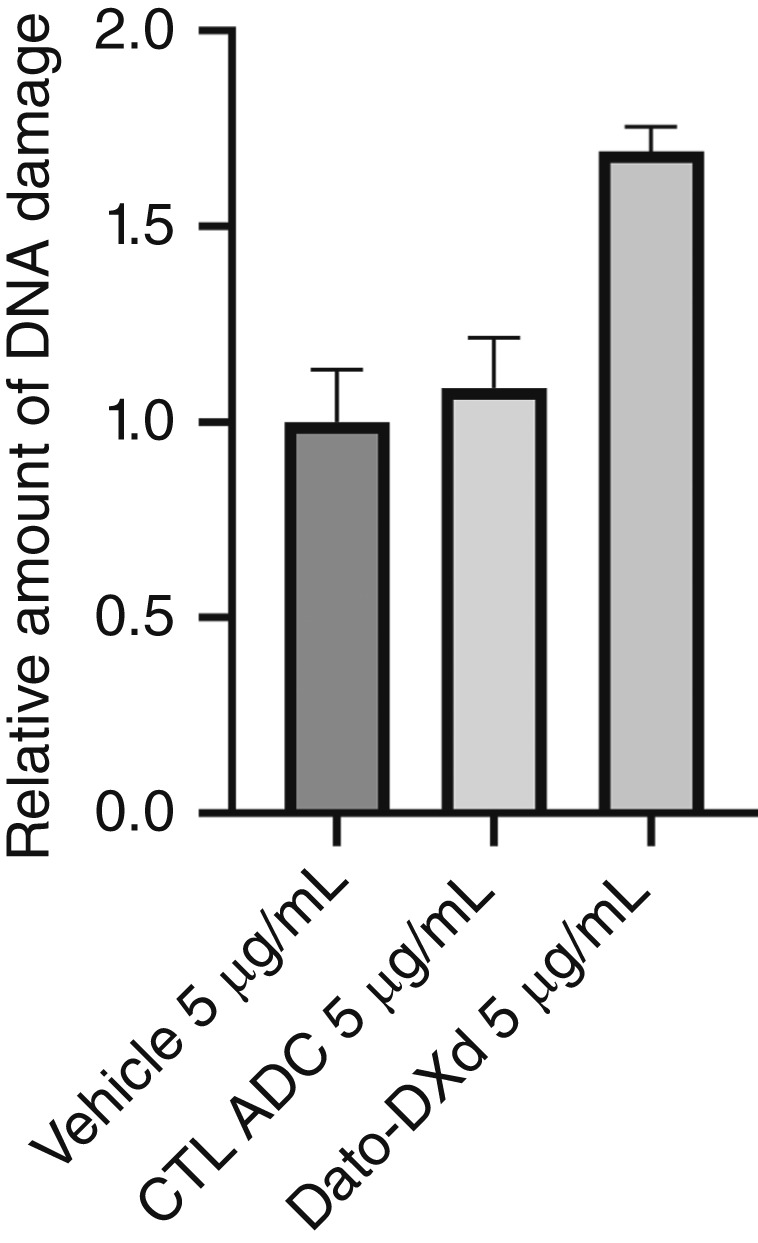
DNA breakage induced from cell internalization of DXd represented by the phosphorylation of H2AX. Greater staining at doses of 5 μg/mL seen with Dato-DXd treatment compared with CTL ADC.

As DNA strand breaks are known to trigger marked phosphorylation of histone H2AX (i.e., γ-H2AX), we took advantage of a standardized assay using a PE mouse Anti-H2AX (pS139) antibody to demonstrate the increased phosphorylation of histone H2AX in the TROP2-positive ARK2 cell line treated with Dato-DXd when compared with the CTL ADC. Dato-DXd induced a significant increase in H2AX phosphorylation when cells were incubated for 72 hours with 5 μg/mL when compared with the treatment with CTL ADC (Fig. 4).

### *In vivo* antitumor activity of Dato-DXd versus CTL ADC

**Figure 5:**
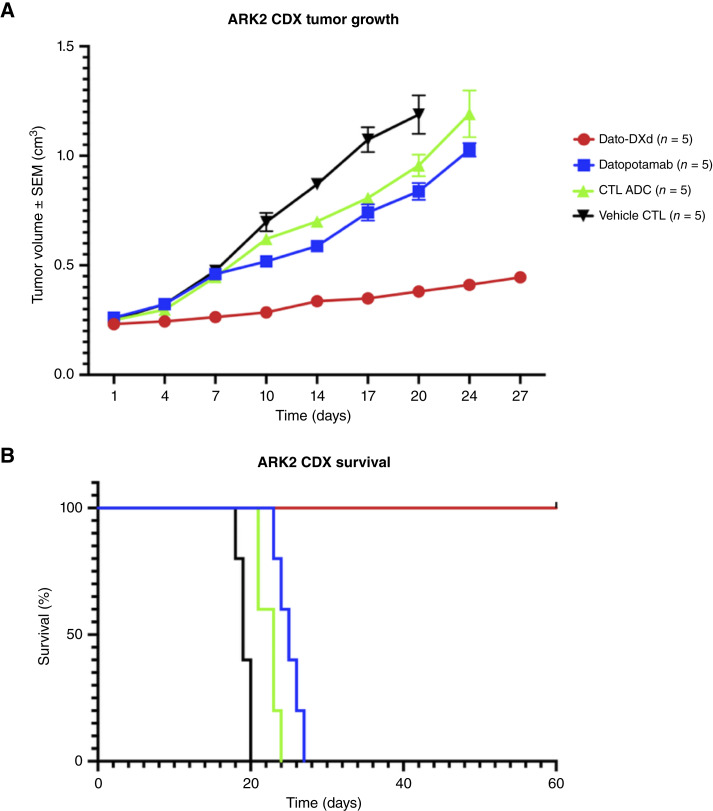
Antitumor activity in mice inoculated with TROP2 3+ USC ARK2 xenograft tumor models (CDXs). **A,** CDX tumor volumes after treatment with Dato-DXd, datopotamab, CTL ADC, and vehicle control (PBS). Dato-DXd demonstrates significant tumor growth inhibition compared with datopotamab, CTL ADC, and vehicle CTL. **B,** OS in CDX after a single retro-orbital treatment with Dato-DXd compared with datopotamab, CTL ADC, and vehicle. OS was significantly prolonged among Dato-DXd–treated groups compared with other treatment groups.

To assess the *in vivo* effects of Dato-DXd, CDXs of a TROP2 3+ USC cell line (ARK2) were used. As outlined in the “Materials and Methods” section, animals were monitored for adequate tumor growth and, upon reaching a tumor size of 0.2 cm^3^, were randomized to treatment groups. Treatment was administered as a single retro-orbital injection of Dato-DXd (day 0). Mice that were randomized to Dato-DXd showed a significant inhibition in tumor growth compared with the alternative three treatment groups (Fig. 5A). At day 7, CDX ARK2 had a mean tumor volume of 0.264 cm^3^ versus 0.459 cm^3^ versus 0.445 cm^3^ versus 0.475 cm^3^ for Dato-DXd, CTL ADC, datopotamab, and vehicle, respectively (*P* < 0.0001 for Dato-DXd vs. CTL ADC on day 7). By day 10, CDX mice treated with Dato-DXd had significantly decreased growth compared with the other three treatments and stable growth since day 7. The datopotamab, CTL ADC, and vehicle groups experienced continued linear growth. At day 24, the Dato-DXd–treated group had a mean tumor size of 0.411 cm^3^, whereas all other treatment groups had mean tumor size in excess of 1 cm^3^, concluding this portion of the experiment. The overall uncorrected Fisher Least Significant Difference test demonstrated significant tumor reduction in Dato-DXd–treated mice compared with datopotamab (*P* < 0.0001), CTL ADC (*P* < 0.0001), and vehicle (*P* < 0.0001) at day 24. For ARK2 TROP3+ CDX mice treated with Dato-DXd, the median OS was significantly improved compared with the other cohorts. The median survival for Dato-DXd was unreached at day 60, as all mice were alive. Conversely, the median survival for datopotamab-treated CDX mice was 25 days, vehicle control was 19 days, and CTL ADC was 23 days. The difference in OS curve between Dato-DXd and three controls was statistically significant (Fig. 5B; i.e., *P* < 0.0001 when comparing Dato-DXd with each control group individually). Animals treated with Dato-DXd did not experience any significant change in body weights or other signs of toxicity.

## Discussion

Endometrial cancer is the most common gynecologic malignancy in the United States with a rapidly changing landscape, including an increased risk of death, now similar to that for ovarian cancer (27). For women who present with early stage type I endometrial cancer (i.e., endometrioid histology), the prognosis is favorable. However, among patients with type II endometrial cancer, such as those diagnosed with USC, there is a high risk for local and distant recurrence even with early stage disease because of the aggressive nature (28). Standard-of-care treatment consists of comprehensive surgical staging or maximal cytoreduction to no residual disease followed by adjuvant chemotherapy with carboplatin and paclitaxel (with the exception of stage IA patients with no residual disease on final pathology) and frequently vaginal cuff brachytherapy (29). The increasing incidence of USC coupled with a disproportionate influence on endometrial cancer–related deaths has led to an increased interest in the tumor biology and mutational patterns in order to identify specific molecular targets for USC (2, 24).

Given the high unmet need for novel targeted therapy for USC, HER2/neu has been identified as the most salient molecular marker (30). Our group as well as others has identified HER2 overexpression in approximately 35% of USC tumors, and it is associated with poor outcomes (31, 32). Notably, HER2 overexpression is more prevalent in African-American patients, who are burdened with an excessive mortality among USC cases (27, 32). Following a successful phase II clinical trial by our group demonstrating the improved PFS and OS for patients with advanced or recurrent HER2-overexpressing USC when treated with trastuzumab in addition to carboplatin/paclitaxel, the National Comprehensive Cancer Network guidelines were revised to routinely include trastuzumab as standard-of-care treatment (33). This has led to further HER2-directed therapies using ADCs, including the use of trastuzumab deruxtecan (T-DXd), a HER2-directed ADC with a toxic Topo I inhibitor payload and high DAR ratio of eight, allowing for serum stability. Following the publication of recently matured data from the DESTINY-PanTumor02, DESTINY-Lung01, and DESTINY-CRC02, the FDA has granted approval for T-DXd in all patients with solid tumor with unresectable or metastatic HER2-positive tumors who have previously been treated without success (13).

The DXd-ADC technology has highly desirable characteristics for an ADC, including an effective payload (DXd), short systemic half-life to minimize off-site toxicity, and stability in circulation reflected in the high DAR (19). Preclinical studies indicate effective use of the DXd payload when targeted at various cancer antigens, indicating the applicability of this payload to be used widely (19–21). This has led to the development of Dato-DXd, an ADC with similar technology to T-DXd, utilizing the same potent Topo I inhibitor and cleavable linker. In this study, we evaluated Dato-DXd using the well-established DXd payload but targeted at TROP2.

Although less well-studied than HER2 in USC, there is growing evidence for the potential of TROP2-targeted therapy in endometrial cancer. When compared with expression in normal tissue, TROP2 is expressed in significantly higher levels in epithelial tumors, making it an excellent target (34). In addition to the recent FDA approval for Dato-DXd, sacituzumab govitecan (SG) is also approved for use in metastatic hormone receptor–positive breast cancer, triple-negative breast cancer, and previously treated metastatic urothelial cancer (26, 35). The toxic payload in SG is SN-38, the active metabolite of irinotecan, which functions as a Topo I inhibitor (34). Although not formally compared in head-to-head preclinical experiments with Dato-DXd, similar drug effectiveness was noted with SG against Trop2-expressing USC cell lines. Additionally, the phase I/II IMMU-123-01 basket trial had favorable outcomes for patients with endometrial cancer (25, 35). This led to NCT04251416, an ongoing open-label phase II trial investigating SG in patients with persistent or recurrent endometrial cancer. Additionally, NCT06040970 is an open-label phase I trial that has not started recruitment yet but is designed to investigate SG use in platinum-sensitive patients with recurrent ovarian and endometrial cancer.

Although preliminary data in solid epithelial cancers and ongoing gynecologic-specific clinical trials indicate that SG is a promising treatment for USC, in terms of pharmacology and tolerance, Dato-DXd has the potential to be an even more effective TROP2 ADC. Dato-DXd has a slightly lower DAR (4 vs. 7.6 in SG) possibly because of its hydrophilic linker, which allows it to retain hydrophilicity and avoid aggregation, leading to improved stability and slower clearance (20, 36, 37). Dato-DXd demonstrates serum stability, with only 5% of the payload released after 21 days, compared with SG, in which 90% is released after 3 days because of linker instability (38). This longer half-life allows for a dosing schedule of every 3 weeks opposed to SG, which is dosed on days 1 and 8 of a 3-week cycle (39). Importantly, the DXd payload is approximately 10 times more potent than irinotecan (40, 41). Although SG is tolerated better than traditional chemotherapy, common side effects include diarrhea and myelosuppression, often requiring the use of antidiarrheal agents and G-CSFs and can lead to dose reductions and treatment interruptions (40). Dato-DXd, in contrast, has a low occurrence of neutropenia, anemia, and diarrhea, with the most common adverse events being nausea and stomatitis (40, 41).

Our results show that Dato-DXd is able to induce significant killing in USC cell lines overexpressing TROP2 (3+). When compared with treatment with CTL ADC, USC cells were highly sensitive to Dato-DXd with a 273.4 and 445 fold decrease in IC_50_ values of ARK2 and ARK20, respectively. In ARK1, a TROP2-nonexpressing cell line, there was no statistically significant difference in the IC_50_ value when treated with Dato-DXd vs. CTL ADC. As such, the *in vitro* cytotoxicity demonstrated is predominately TROP2-mediated. Through the ADCC assay, our results show that Dato-DXd successfully causes apoptosis in TROP2-overexpressing USC cell lines. These data with Dato-DXd are consistent with our recent results using T-DXd in USC in which strong ADCC was demonstrated in the presence of effector cells, suggesting that the toxic payload of the ADC may only partially interfere with the binding of the Fc receptor of NK cells and their consequent activation, as demonstrated by the similar levels of ADCC we observed in our 4-hour chromium release assay when compared with the unconjugated datopotamab antibody (42).

Intratumoral heterogeneity poses a known challenge when using highly specific target drugs such as ADCs. Therefore, the ability of an ADC to demonstrate bystander antitumor effect is a mandatory requirement in gynecologic tumors, which are known to demonstrate heterogeneity in target expression. Following the internalization of Dato-DXd by tumor cells, DXd is cleaved from its linker by lysosomal enzymes, including cathepsins, and the payload is able to permeate through cell membranes into adjacent tumor cells regardless of antigen expression. This results in killing of tumor cells regardless of TROP2 expression, making the ADC effective in heterogeneously expressing TROP2 tumors. To validate this hypothesis, we admixed a TROP2-nonexpressing cell line (ARK4) with a TROP2-overexpressing cell line (ARK2) and were able to demonstrate greater killing in the cocultured cells treated with Dato-DXd than with CTL ADC. Although in the breast cancer study TROPION-B01 there was no association between Trop2 expression levels and clinical response to Dato-Dxd in patients, our study results demonstrating a correlation between TROP2 expression and preclinical activity are in agreement with previous preclinical work with other ADCs (i.e., SG) targeting TROP2 (43).

Importantly, *in vivo* experiments in xenograft mice models with TROP2 overexpression treated with Dato-DXd experienced tumor regression and prolonged survival compared with xenografts treated with datopotamab, CTL ADC, or vehicle.

In conclusion, TROP2 overexpression is frequently encountered in biologically aggressive USC tumors. USC cell lines overexpressing TROP2 are susceptible to killing by Dato-DXd, evident in our cytotoxicity assays and ADCC-mediated killing experiments. Dato-DXd exhibits the bystander antitumor effect by successfully killing adjacent cells regardless of TROP2 expression. Finally, mouse xenograft models were highly sensitive to Dato-DXd *in vivo*. Taken together, these experiments demonstrate that Dato-DXd has significant preclinical activity in USC with TROP2 overexpression. On the basis of these results, future clinical trials in patients with TROP2-overexpressing USC are warranted.
